# Deregulation in adult IgA vasculitis skin as the basis for the discovery of novel serum biomarkers

**DOI:** 10.1186/s13075-024-03317-6

**Published:** 2024-04-12

**Authors:** Matija Bajželj, Matjaž Hladnik, Rok Blagus, Vesna Jurčić, Ana Markež, Tanya Deniz Toluay, Snežna Sodin-Šemrl, Alojzija Hočevar, Katja Lakota

**Affiliations:** 1 Department of Rheumatology, University Medical Centre Ljubljana, Ljubljana, Slovenia; 2 Faculty of Mathematics, Natural Sciences and Information Technologies, University of Primorska, Koper, Slovenia; 3 Faculty of Medicine, University of Ljubljana, Ljubljana, Slovenia; 4 Institute for Biostatistics and Medical Informatics, Faculty of Medicine, University of Ljubljana, Ljubljana, Slovenia; 5 Institute of Pathology, Faculty of Medicine, University of Ljubljana, Ljubljana, Slovenia; 6 Master Study of Applied Statistics, Faculty of Electrical Engineering, University of Ljubljana, Ljubljana, Slovenia

**Keywords:** IgA vasculitis, Adults, RNA sequencing, Lipid metabolism, Acute inflammatory response, Serum biomarkers, Machine learning

## Abstract

**Introduction:**

Immunoglobulin A vasculitis (IgAV) in adults has a variable disease course, with patients often developing gastrointestinal and renal involvement and thus contributing to higher mortality. Due to understudied molecular mechanisms in IgAV currently used biomarkers for IgAV visceral involvement are largely lacking. Our aim was to search for potential serum biomarkers based on the skin transcriptomic signature.

**Methods:**

RNA sequencing analysis was conducted on skin biopsies collected from 6 treatment-naïve patients (3 skin only and 3 renal involvement) and 3 healthy controls (HC) to get insight into deregulated processes at the transcriptomic level. 15 analytes were selected and measured based on the transcriptome analysis (adiponectin, lipopolysaccharide binding protein (LBP), matrix metalloproteinase-1 (MMP1), C-C motif chemokine ligand (CCL) 19, kallikrein-5, CCL3, leptin, C-X-C motif chemokine ligand (CXCL) 5, osteopontin, interleukin (IL)-15, CXCL10, angiopoietin-like 4 (ANGPTL4), SERPIN A12/vaspin, IL-18 and fatty acid-binding protein 4 (FABP4)) in sera of 59 IgAV and 22 HC. Machine learning was used to assess the ability of the analytes to predict IgAV and its organ involvement.

**Results:**

Based on the gene expression levels in the skin, we were able to differentiate between IgAV patients and HC using principal component analysis (PCA) and a sample-to-sample distance matrix. Differential expression analysis revealed 49 differentially expressed genes (DEGs) in all IgAV patient’s vs. HC. Patients with renal involvement had more DEGs than patients with skin involvement only (507 vs. 46 DEGs) as compared to HC, suggesting different skin signatures. Major dysregulated processes in patients with renal involvement were lipid metabolism, acute inflammatory response, and extracellular matrix (ECM)-related processes. 11 of 15 analytes selected based on affected processes in IgAV skin (osteopontin, LBP, ANGPTL4, IL-15, FABP4, CCL19, kallikrein-5, CCL3, leptin, IL-18 and MMP1) were significantly higher (p-adj < 0.05) in IgAV serum as compared to HC. Prediction models utilizing measured analytes showed high potential for predicting adult IgAV.

**Conclusion:**

Skin transcriptomic data revealed deregulations in lipid metabolism and acute inflammatory response, reflected also in serum analyte measurements. LBP, among others, could serve as a potential biomarker of renal complications, while adiponectin and CXCL10 could indicate gastrointestinal involvement.

**Supplementary Information:**

The online version contains supplementary material available at 10.1186/s13075-024-03317-6.

## Introduction

Immunoglobulin A vasculitis (IgAV) is an immune complex leukocytoclastic vasculitis characterized by the dominant deposition of immunoglobulin (Ig) A in the affected vascular wall [1]. Cutaneous vasculitis in a form of palpable purpura represents the hallmark of the disease that frequently affects also other organs - kidneys, joints and gastrointestinal (GI) tract. In adults commonly a histological confirmation is needed for diagnosis [2]. Typical findings in skin biopsies are the infiltration of blood vessel walls and adjacent interstitium with granulocytes, predominantly neutrophils, fibrin deposition, and the presence of IgA complexes in vessel walls [1]. Later, mononuclear inflammatory cell-rich infiltrates, containing macrophages and lymphocytes, gradually replace neutrophils [3]. Skin involvement in adult IgAV has mainly been investigated using histological techniques, focusing on cellular and tissue imaging. Two studies investigated the expression pattern of miRNAs in adult IgAV [4, 5], while studies performing mRNA profiling of the affected skin from adult IgAV patients are still lacking.

While IgAV might be self-limiting in some adults, others could have severe, even life-threatening visceral involvement. GI involvement (IgAV-GI) may manifest with a severe haemorrhage or bowel perforation, and renal involvement (IgAVN) can progress to acute kidney injury and chronic kidney failure [6]. Nevertheless, diagnostic and predictive biomarkers of systemic and organ-specific involvement in acute adult IgAV have not been extensively studied. Few studies reported patient age forecasts of visceral involvement – as younger patients have more frequent GI and joint involvement, while older patients more often present with renal disease [7]. Hočevar et al. identified that more extensive skin lesions at presentation and elevated pre-treatment neutrophil-to-lymphocyte ratio (NLR) predicted both renal and GI involvement (IgAVN + GI), and active smoking was associated with IgAVN [8]. Only few studies analysed serum in IgAV, reporting an increase in acute-phase reactants, such as C-reactive protein (CRP) and serum amyloid A (SAA) [9, 10] as well as proinflammatory cytokines, such as interleukin (IL)-6, IL-8, IL-1β and tumor necrosis factor (TNF)-α [9, 11] and increased plasma concentrations of C-X-C motif chemokine ligand (CXCL) 9, CXCL13 and CXCL10/11 [12]. Appropriate biomarkers might support clinicians more optimally in identifying IgAV patients before skin biopsy results are available, thus facilitating treatment decisions.

Our first aim was to study deregulated biological processes in adult IgAV patients’ skin at the mRNA level. Our second aim was to find potential associations between skin lesions and systemic disease. Based on the skin mRNA signature, we selected analytes to be measured in patient’s sera using multiplex assay. Machine learning was used to study the ability of measured analytes in predicting adult IgAV phenotype. Exploratory analysis of available skin histopathological findings was performed and correlations with serum analytes were investigated.

## Methods

### Study design, study subjects and clinical data collection

Adults (aged ≥ 18) with IgAV, diagnosed for the first time between February 1, 2015 and July 30, 2022, participated in the unicenter study at the University Medical Center Ljubljana (UMCL). Patients’ data were collected in a prospective manner. All patients underwent an extensive clinical evaluation and laboratory workup. All subjects signed informed consent. The study was approved by the Slovene National Medical Ethics Committee (#159/07/13, #99/04/15, #65/01/17 and #0120–121/2021/3).

In the study, we used the definitions of purpura above waistline, GI and renal involvement, as previously reported in detail [13]. The diagnosis of IgAV was established according to the 2012 revised International Chapel Hill Consensus Conference Nomenclature of Vasculitides [1]. All included IgAV patients fulfilled the 2010 European League Against Rheumatism/Paediatric Rheumatology International Trials Organisation/Paediatric Rheumatology European Society (EULAR/PRINTO/PRES) classification criteria and had histologically-proven disease [14].

Clinical data collected were: age, sex, body mass index (BMI), smoking status, duration of clinical symptoms, past medical history, past and concurrent infections, IgAV-related signs and symptoms. Disease activity was determined using Birmingham vasculitis activity score (BVAS). Healthy controls (HC) were biopsied solely for the needs of this study and were investigated at the Department of Rheumatology, UMCL for the presence of systemic autoimmune rheumatic diseases (SARDs) and immunosuppressive treatment as an exclusion criteria.

### Laboratory parameters

Erythrocyte sedimentation rate (ESR), CRP, complete blood count with differential, basic biochemistry panels including electrolytes, creatinine, urea, serum protein electrophoresis, serum IgA, M, G levels, complement components C3 and C4 and urine analysis were determined for routine diagnostics. The concentration of SAA was determined using immunonephelometry (BN Prospec System, Siemens).

### RNA extraction, sequencing and bioinformatic analysis

Six adult treatment-naïve IgAV patients and three healthy controls were consecutively recruited for RNA sequencing of skin biopsies (Additional file 2: Table S1). Skin biopsy samples from the shin were collected during the diagnostic procedure. Samples were immediately stored in Allprotect Tissue Reagent (Qiagen, Germantown, MD, USA) and kept at -80^o^C untill the isolation process. RNA from the skin biopsy samples was isolated using TissueLyser LT (Qiagen, Germantown, MD, USA) and RNeasy Mini Kit (Qiagen, Germantown, MD, USA) according to the manufacturer’s protocol. The quantity and quality of the RNA obtained were checked using NanoDrop 2000 spectrophotometer (Thermo Fisher Scientific, Waltham, MA, USA).

The isolated RNA from skin-biopsy samples was used for RNA-seq library preparation with TruSeq RNA Access Library Prep Kit (Gold, Ilumina, San Diego, CA, USA). Paired-end Sequencing, 2 × 100 bp, was performed using Illumina HiSeq 4000 platform (25.9 to 30.8 million reads were obtained per sample). For analysis, FastQC v0.11.9 was used to perform quality control analysis, followed by Trimmomatic 0.36 (using “snakemake wrapper 0.77.0/bio/trimmomatic/pe”) to remove adapter sequences. The expression of transcripts was quantified with Salmon v1.5.2 using the mapping-based model. Full decoy salmon index was prepared with the transcriptome (cDNA.all.fa and ncrna.fa) and genome reference files of Ensembl release 104. The quantification of paired-end reads was performed with options validateMappings, seqBias, and gcBias. Salmon quantification files were imported into R using tximeta (v1.8.5) package and transcript-level quantification was summarized to gene-level. Genes with less than 10 counts over all samples included in the analysis were removed prior to differential gene expression analysis with DESeq2 package (v1.30.1) (http://bioconductor.org/packages/DESeq2) to compare skin samples from IgAV-with renal involvement (IgAVN) (*n* = 3) with patients with skin-limited disease (sl-IgAV) (*n* = 3) and age-and sex-matched healthy controls (HC) (p values were obtained with Wald test and adjusted p values were calculated using the Benjamini and Hochberg procedure). Shrinkage estimator method, apeglm (approximate posterior estimation for GLM), was used with lfcShrink DESeq2 function to estimate the log-fold changes [15]. Differentially expressed genes were defined with shrunken log2 fold change ≥ │1│, p-adj < 0.05. In order to investigate whether it is possible to discriminate between patients’ subgroups and HC, a principal component analysis (PCA) was performed using plot PCA function, which considered the 500 most variable genes. Additionally, hierarchical clustering with heatmap of sample-to-sample distances was prepared. This was based on the Euclidean distances and employed the complete-linkage agglomeration method, utilizing the dist and pheatmap (from the pheatmap package) functions. For both analyses (PCA and hierarchical clustering), count data was transformed using the regularized logarithm (via the DESeq2 rlog function). Gene set enrichment analysis (GSEA) and over-representation analysis (ORA) of Kyoto Encyclopedia of Genes and Genomes (KEGG) and Gene Ontology (GO) analysis for biological processes (BP) and cellular components (CC) was performed with R package clusterProfiler (https://bioconductor.org/packages/release/bioc/html/clusterProfiler.html). The Human Protein Atlas database was used to select proteins appropriate for the multiplex serum analysis.

### Luminex assay

Fifty-nine treatment-naïve adult IgAV patients (41 males, 18 females, median (IQR) age 64.8 (49.4–70.3 years) were recruited for the serum analytes measurement (Additional file 3: Table S2). HC comprised of 22 age and sex matched individuals (Additional file 3: Table S2).

After blood withdrawal, blood clotting was allowed for 30 min and samples centrifuged at 3000×g for 5 min, sera were aliquoted, and stored at − 20 °C until further analysis. Luminex assay was performed in sera from 59 treatment-naïve adult IgAV patients and 22 age-/sex-matched HC. Concentrations of 15 serum analytes from IgAV patients and HC (adiponectin, lipopolysaccharide binding protein (LBP), matrix metalloproteinase-1 (MMP1), C-C motif chemokine ligand (CCL) 19, kallikrein-5, CCL3, leptin, CXCL5, osteopontin, IL-15, CXCL10, angiopoietin-like 4 (ANGPTL4), SERPIN A12/vaspin, IL-18 and fatty acid-binding protein 4 (FABP4)) were measured by the Luminex platform (MAGPIX System, Merck, Darmstadt, Germany) using human pre-mixed multi-analyte kits (R&D Systems) according to manufacturer instructions.

### Histopathology and immunofluorescence

Skin biopsies from treatment-naïve patients were evaluated using light microscopy and direct immunofluorescence. Detailed histopathological and immunofluorescence findings were provided for 34 out of 59 patients that were included in the analyte measurements. For light microscopy, skin tissue samples were fixed in 10% neutral buffered formalin and embedded in paraffin. Paraffin blocks were selected to include skin samples of at least 4 mm in length and 2 mm in depth. Tissue sections were stained with haematoxylin and eosin, and phosphotungstic acid–haematoxylin to demonstrate fibrinoid necrosis. Intensities of inflammatory infiltrates (separately for granulocytes and mononuclear inflammatory cells) involving small vessels in the dermis were evaluated. The presence of fibrinoid necrosis of vessels was assessed as absent (0) or present (1). For immunofluorescence microscopy, skin samples were frozen in liquid nitrogen, and cryostat sections were stained with fluorescein isothiocyanate-labelled antisera against human IgA, IgG, IgM, C3, C1q, and fibrin/fibrinogen (Dako, Copenhagen, Denmark). The intensity of immune complexes was semi quantitatively assessed as mild or abundant.

### Statistical analysis

Summary statistics are expressed as medians and 25–75th percentiles (Q25–Q75) with Luminex measurements logarithmically transformed. PCA was performed to distinguish between patients and HC based on measurement of all 15 Luminex analytes. Differences between the two groups were determined by the Mann Whitney U Test. All p-values were adjusted for multiple comparisons using Benjamini and Hochberg method to control the false discovery rate. The linear regression model was used to investigate whether the differences between the two groups for 15 measured analytes persist even after correcting for age and sex. To predict IgAV based on 15 measured analytes, two predictive models, ST-EasyEnsemble [16] and random forest [17], were built. To assess the predictive value of each model we calculated the area under the ROC curve (AUC). The AUC was calculated by splitting the data into a training (80%) and test set (20%) repeating the procedure 100 times (the results are averaged over 100 repetitions). For each model, the relative importance of variables was calculated and expressed as percentages. The ten most important variables were presented graphically. The heatmap.2 function from the gplots package was used to visualize Pearson correlation coefficients of the correlation matrices. The analysis was performed in R language for statistical computing (R version 4.0.5 [18]) and GraphPad Prism version 9.0. Adjusted P-values of < 0.05 were regarded as statistically significant.

## Results

### Comparison of the skin transcriptomic profiles from all IgAV patients, IgAVN, sl-IgAV and HC

Among six samples used for RNAseq three patients had skin-limited IgAV (sl-IgAV) and three IgAVN (Additional file 2: Table S1). All patients and HC except 1 were females, and similar age (median (IQR) of 66.5 (56.6–76.7). Considering extension of skin lesions in both patient groups (skin limited IgAV and IgAVN) purpura above waistline was present in 2 out of 3 patients and 1 patient in each group had skin necroses, while bullous lesions were seen in 1 patient in skin-limited IgAV group. Median duration of symptoms in both groups was similar (5 vs. 6 days, respectively). While in sl-IgAV group one patient had recent infection (skin infection) and one had concurrent infection (urinary tract infection), there were 2 patients with recent infection in IgAVN group (both patients had upper respiratory tract infection). All three IgAVN patients had microhaematuria and proteinuria was seen in 2 patients (0.5 g/day and 1.0 g/day). None of them had acute kidney injury. sl-IgAV and IgAVN patients included in the RNA sequencing of skin biopsies did not have gastrointestinal or joint involvement.

**Fig. 1:**
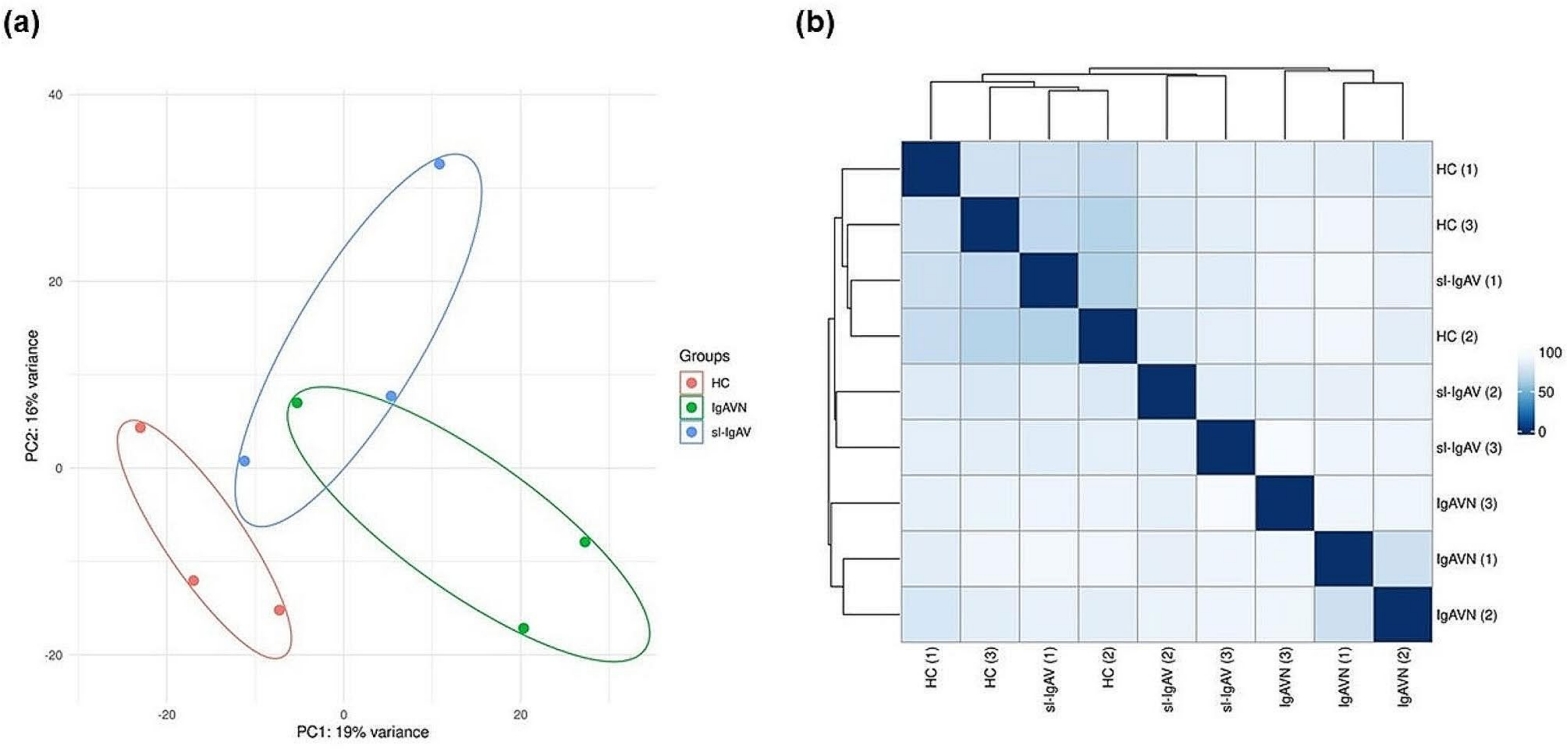
PCA (a) and hierarchical clustering of subjects with heatmap showing subject-to-subject distances (b). PCA and subject clustering were performed with the gene expression count data, previously transformed with the regularized logarithm (as implemented in the DESeq2 rlog function). **(a)** PCA distinguished two patient groups from HC. Each point on the plot represents an individual sample, as HC are in red, IgAVN patients in green and sl-IgAV patients in blue. Samples that cluster closer together on the plot share more similar gene expression patterns. **(b)** Hierarchical clustering of subjects, utilizing the Euclidean distance and the complete-linkage agglomeration method, revealed distinct transcriptomes for IgAVN, sl-IgAV and HC. However, the subject sl-IgAV [1] was an exception, clustering with the HC subjects instead. Each row and column correspond to a different individual. The matrix depicts the degree of similarity or dissimilarity between pairs of samples based on their gene expression profiles. Darker colours or shorter distances indicate greater similarity, while brighter colours or longer distances represent increased dissimilarity. PCA, principal component analysis; IgAV, Immunoglobulin A vasculitis; HC, healthy controls; IgAVN, IgA with renal involvement; sl-IgAV, skin-limited IgAV.

Principal component analysis (PCA) and sample-to-sample distance matrix showed that IgAVN, sl-IgAV patients and HC can be distinguished (Fig. 1). Differential expression analysis revealed 49 differentially expressed genes (DEGs) in all IgAV patients vs. HC, with majority of genes being upregulated. Patients with IgAVN had 507 DEGs while patients with sl-IgAV only 46 DEGs when compared to HC. A direct comparison of DEGs between IgAVN and sl-IgAV patients confirmed the difference in transcriptomes with 136 DEGs (Additional file 4: Table S3).

### Analysis of deregulated biological processes in IgAV skin—pathway enrichment analysis

**Fig. 2:**
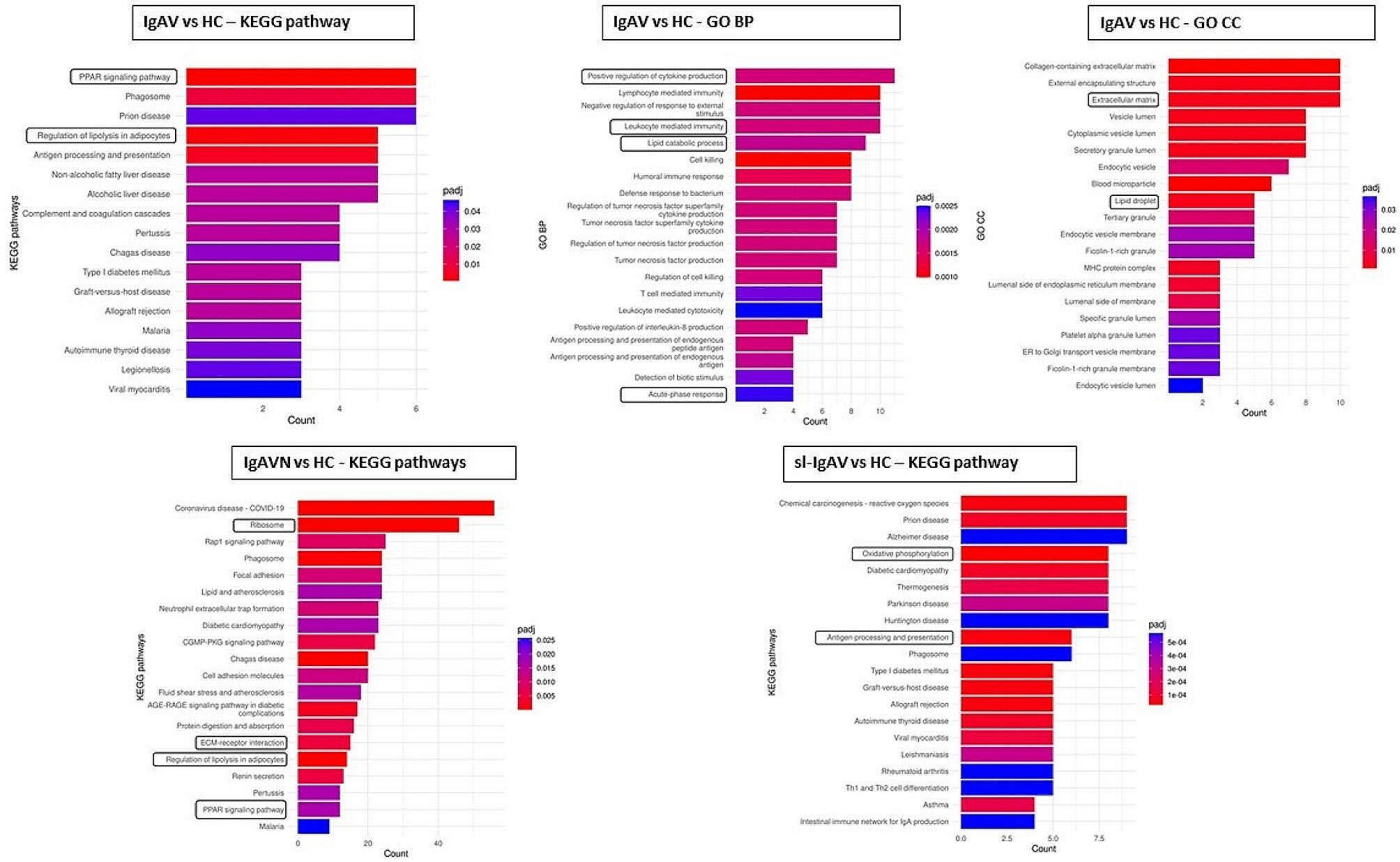
Over-Representation analysis (ORA) of enriched KEGG pathways, GO BP and CC in IgAV patients vs. HC, IgAVN vs. HC and sl-IgAV vs. HC performed on the gene sets derived from RNA sequencing data. Each bar on the graph represents a biological term, such as KEGG pathway, BP, or CC. Enriched terms are color-coded, with red colors indicating higher significance. The level of statistical significance is expressed with p-adj values. Hypergeometric test was employed to determine if the observed enrichment exceeded what might be expected by chance. The adjusted p value was computed using the Benjamini-Hochberg procedure, and a q value threshold of 0.05 was set to deem enriched terms as significant. The x-axis shows gene count. KEGG, Kyoto Encyclopedia of Genes and Genomes; GO, Gene Ontology; BP, Biological Processes; CC, Cellular Components; IgAV, immunoglobulin A vasculitis; HC, healthy controls; IgAVN, IgAV with renal involvement; sl-IgAV, skin-limited IgAV.

Over-representation analysis (ORA) of KEGG (Kyoto Encyclopedia of Genes and Genomes) Pathways revealed PPAR (peroxisome proliferator-activated receptors) signalling pathway (p-adj = 5.533 × 10^− 4^) and Regulation of lipolysis in adipocytes (p-adj 1.3570 × 10^− 3^) among most enriched pathways in all IgAV compared with HC. Among the most enriched GO Biological Processes (BP) were positive regulation of Cytokine production (1.25 × 10^− 3^), Leukocyte mediated immunity (1.25 × 10^− 3^), Lipid catabolic process (p-adj = 1.80 × 10^− 3^) and Acute-phase response (p-adj = 2.46 × 10^− 3^). For the most enriched GO cellular components (CC) we identified Extracellular matrix (ECM) (p-adj = 2.62 × 10^− 3^) and Lipid droplet (p-adj = 2.76 × 10^− 3^). In the IgAVN group as compared to HC ORA revealed the enrichment of Ribosome (p-adj < 1 × 10^− 7^), Regulation of lipolysis in adipocytes (p-adj = 2.25 × 10^− 4^) and ECM-receptor interaction (p-adj = 4.80 × 10^− 3^) KEGG pathways. On the other hand, sl-IgAV versus HC exhibited enriched KEGG pathways Oxidative phosphorylation (p-adj = 2.74 × 10^− 5^) and Antigen processing and presentation (p-adj = 4.06 × 10^− 5^), suggesting differences between IgAVN and sl-IgAV on molecular level (Fig. 2). ORA did not reveal any enriched terms for the comparison IgAVN vs. sl-IgAV, while complementary approach, gene set enrichment analysis (GSEA) revealed the enrichment of ECM organization (p-adj = 8.37 × 10^− 4^) and Ribosome biogenesis (p-adj = 8.37 × 10^− 4^) GO BP (Additional file 1: Figure S1). Biological interpretation of RNA sequencing data suggested most prominent deregulation in lipid metabolism, acute inflammatory response and ECM in IgAV skin.

### Analysis of serum analytes profiles

Based on the deregulated processes we identified hub genes and investigated if their protein levels could reflect differences between IgAV subgroups and HC. Eight analytes were among DEGs in IgAVN as compared to HC (Additional file 1: Figure S2) and belonged to processes lipid metabolism (adiponectin, leptin, ANGPTL4, SERPIN A12 and FABP4), acute phase response (LBP) and ECM (MMP1 and osteopontin). The other 7 analytes were associated with GO BP Positive regulation of cytokine production and Leukocyte mediated autoimmunity.

Serum sample of 59 treatment-naïve adult IgAV patients were included. Median (IQR) patient age was 64.8 (49.4–70.3) years and median (IQR) symptom duration time before blood samples were taken was 7 (5–14) days. Eleven (18.6%) patients reported constitutional symptoms (36.4% fever and 72.7% weight loss). While all patients had skin involvement (66% of them had purpura above waistline and 50% necrotic skin lesions), 21 (35.6%) patients had skin limited disease, and the others systemic disease. Nineteen (32.2%) had renal involvement based on urinalysis (glomerular haematuria, proteinuria, presence of red cell casts). Twelve (20.3%) patients had concurrent GI and renal involvement, while 7 (11.9%) had GI involvement (85.7% abdominal pain, 57% diarrhoea, 85.7% gastrointestinal bleeding, 28.6% ileus). Articular involvement was additionally seen in 8 patients (13%; 100% arthralgias; 25% arthritis).

**Fig. 3:**
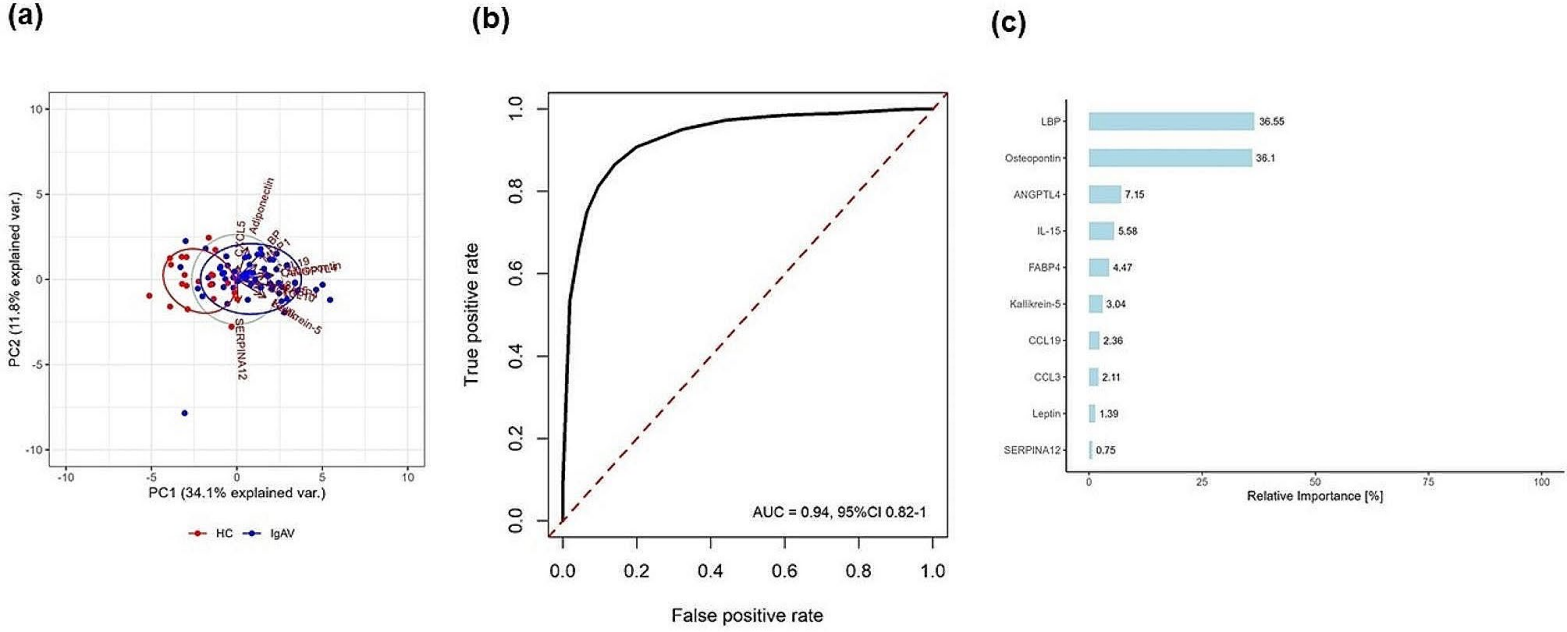
Classification of adult IgAV using Random Forest **(a)** Principal component analysis of serum analyte concentrations distinguished IgAV patients and HC. **(b)** ROC curve of Random forest algorithm with corresponding AUC (with 95% confidence interval). Red line presents ROC curve of a random classifier (AUC = 0.5). **(C)** First ten variables according to their relative importance for prediction of adult IgAV. IgAV, Immunoglobulin A vasculitis; HC, healthy controls; PC, principal component; ROC, Receiver operating characteristic; AUC, area under the curve; LBP, Lipopolysaccharide binding protein; ANGPTL4, angiopoietin-like 4; IL, interleukin; FABP4, fatty acid-binding protein 4; CCL, Chemokine (C-C motif) ligand

Among the 15 measured analytes, levels of 11 analytes (osteopontin, LBP, ANGPTL4, IL-15, FABP4, CCL19, kallikrein-5, CCL3, leptin, IL-18 and MMP1) were significantly higher (p-adj < 0.05) in treatment-naïve adult IgAV patients as compared to HC. These differences could not be explained by potential age- and sex-difference between IgAV and HC as suggested by the age- and sex-adjusted analysis. The highest increase in serum median levels of IgAV patient’s vs. HC were observed for kallikrein-5 (5.97-fold change), osteopontin (4.25-fold), FABP4 (2.78-fold), and LBP (2.35-fold) (Table 1). PCA suggested that IgAV patients can potentially be distinguished from the HC (Fig. 3a). Random Forest (RF) based on measured analytes had good predictive ability for the IgAV prediction, with out-of-sample area under the receiver operating characteristic (ROC) curve (AUC) 0.94 (95% confidence interval (CI) 0.82-1.00) (Fig. 3b). The two most important variables identified by RF were LBP and osteopontin (Fig. 3c). Similar results were obtained with ST-EasyEnsemble (Additional file 1: Figure S2).

**Table 1:** Measured protein analytes in sera of adult IgAV patients as compared to HC

	Median (Q_25_-Q_75_)				
Serum analyte	IgAV (*n* = 59)	HC (*n* = 22)	p-adj value (Mann-Whitney U test)	p-adj (linear regression adjusted for age and sex)	Fold change
Adiponectin	6.08 (4.59–7.73)	5.54 (4.42–7.24)	5.92e^− 1^	7.80e^− 1^	1.10
**LBP**	14.63 (9.90–20.3)	6.23 (4.9–7.71)	1.43e^− 6^	**1.79e** ^**− 3**^	2.35
**MMP1**	4.46 (2.57–8.56)	1.47 (3.07–4.75)	9.09e^− 3^	**3.86e** ^**− 3**^	1.45
**CCL19**	123.4 (79.8-196.2)	73.9 (35.2–93.9)	1.20e^− 3^	**1.41e** ^**− 3**^	1.67
**kallikrein-5**	309.4 (137.3-559.8)	51.8 (4.50–224.0)	1.20e^− 3^	**9.87e** ^**− 4**^	5.97
**CCL3**	290.9 (243.6-378.5)	214.6 (129.2-288.3)	2.59e^− 3^	**4.37e** ^**− 3**^	1.36
**leptin**	9.15 (5.64–18.5)	4.80 (2.21–6.81)	2.84e^− 3^	**5.60e** ^**− 3**^	2.35
CXCL5	1.34 (1-02-1.99)	1.42 (0.99–2.02)	8.28e^− 1^	8.77e^− 1^	0.950
**Osteopontin**	42.8 (30.1–77.9)	10.1 (6.65-20.0)	4.20e^− 7^	**2.73e** ^**− 8**^	4.25
**IL-15**	3.29 (4.91–5.72)	6.52 (5.72–9.72)	2.32e^− 5^	**3.19e** ^**− 7**^	1.33
CXCL10	32.1 (22.5–48.6)	25.8 (20.7–36.9)	2.16e^− 1^	1.22e^− 1^	1.24
**ANGPTL4**	217.6 (132.9-312.9)	113.2 (88.4–131.0)	1.79e^− 5^	**3.13e** ^**− 7**^	1.92
SERPIN A12	0.2 (0.1–0.33)	0.22 (0.1–0.64)	4.40e^− 1^	2.40e^− 1^	0.902
**IL-18**	0.17 (0.12–0.23)	0.12 (0.10–0.15)	3.45e^− 3^	**5.60e** ^**− 3**^	1.47
**FABP4**	44.6 (21.1–77.9)	16.0 (10.2–20.8)	3.74e^− 5^	**2.67e** ^**− 7**^	2.78

IgAV, immunoglobulin A vasculitis; HC, healthy controls; p-adj, p-adjusted value; LBP, lipopolysaccharide binding protein; MMP1, matrix metalloproteinase-1; CCL, C-C motif chemokine ligand, CXCL, C-X-C motif chemokine ligand; IL, interleukin; ANGPTL4, angiopoietin-like 4; FABP4, fatty acid-binding protein 4

### Associations of measured analytes with clinical manifestations

**Fig. 4:**
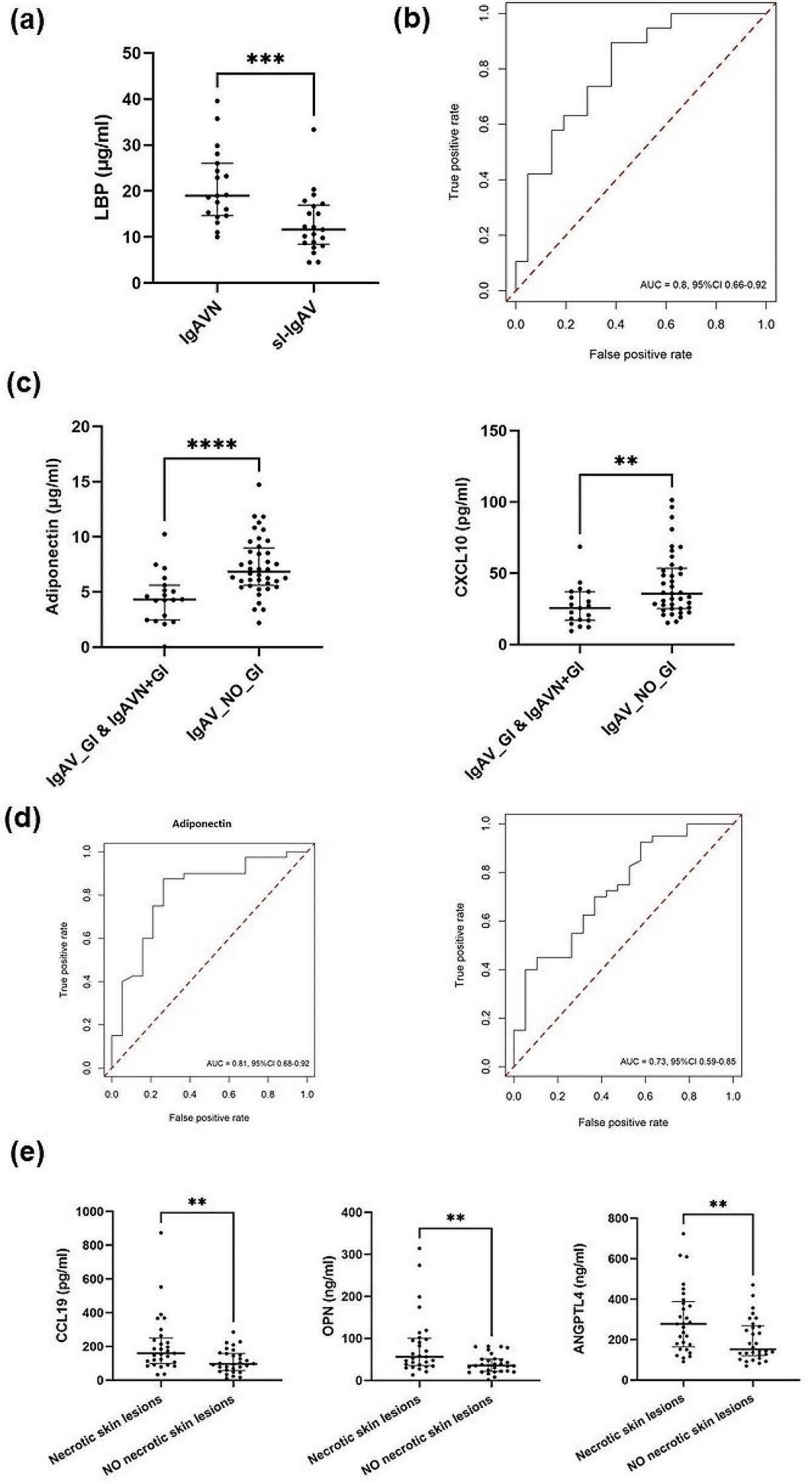
Measured analytes distinguishing between sl-IgAV, IgAVN and IgAV_GI. **(a)** LBP concentration was significantly higher in IgAVN as compared to sl-IgAV patients as calculated using Mann Whitney U Test. **(b)** ROC curve of LBP for predicting renal involvement. **(c)** Serum levels of adiponectin and CXCL10 were significantly lower in all patients with GI involvement (IgAV_GI & IgAVN + GI) as compared to those without. **(d)** ROC curve of adiponectin and CXCL10 for predicting renal involvement. **(e)** Serum levels of CCL19, osteopontin and ANGPTL4 were significantly elevated in IgAV patients with necrotic skin lesions. P-values were calculated using Mann Whitney U Test. Data are expressed as medians (Q25-Q75) of each group. *P ≤ 0.05, **P ≤ 0.01, ***P ≤ 0.001. PC, principal component; ROC, Receiver operating characteristic; IgAV, immunoglobulin A vasculitis; sl-IgAV, skin-limited IgAV; IgAVN, IgAV with renal involvement; IgAV_GI, IgA with gastrointestinal involvement (GI); IgAVN + GI, IgAV with GI and renal involvement; HC, healthy controls; LBP, Lipopolysaccharide binding protein; CXCL, C-X-C motif chemokine ligand; CCL, Chemokine (C-C motif) ligand; ANGPTL4, angiopoietin-like 4

Renal and GI involvement represent major complications in IgAV patients, thus associations with respective organ involvement were investigated. LBP was significantly higher (p-adj = 1.16 × 10^− 2^) in IgAVN as compared to sl-IgAV (Fig. 4a) with corresponding AUC 0.80 (95% CI 0.65–0.93) (Fig. 4b). The other two acute phase reactants, CRP (p-adj = 0.14) and SAA (p-adj = 0.20) were also increased in IgAVN, however not to significant levels. All patients with GI involvement (IgAV_GI & IgAVN + GI) had significantly lower serum levels of adiponectin (p-adj = 2.07 × 10^− 3^) and CXCL10 (3.57 × 10^− 2^) (Fig. 4c) with corresponding AUC 0.81 (95% CI 0.69–0.93) and AUC 0.73 (95% CI 0.59–0.87) respectively (Fig. 4d). PCA indicated that those patients can be distinguished from patients without GI involvement (Additional file 1: Figure S3a). RF achieved out-of-sample AUC 0.68 (95% CI 0.32–0.96) to predict GI involvement based on 15 measured analytes (Additional file 1: Figure S3b). Adiponectin and CXCL10 were identified as the most important variables for predicting GI involvement (Additional file 1: Figure S3c). Similar result with AUC 0.75 was obtained with ST-EasyEnsemble (Additional file 1: Figure S4). We also investigated associations between measured analytes and skin lesions. CCL19 (p-adj = 3.68 × 10^− 2^), osteopontin (p-adj = 3.68 × 10^− 2^) and ANGPTL4 (4.47 × 10^− 2^) were significantly higher in IgAV patients with necrotic purpura (Fig. 4e).

### Associations of measured analytes with routine laboratory data

Results of routine laboratory tests are presented in Additional file 3: Table S2. Serum levels of IgA were elevated above 4g/l in 32/59 (54%) patients. Inflammatory markers including ESR, CRP and SAA medians were above the normal range. Significant correlation coefficients (r_s_) in IgAV patients were observed between individual analytes measured in our study (Additional file 1: Figure S5), namely CRP positively correlated with LBP (r_s_ = 0.617, p-adj = 9.55 × 10^− 6^) and ANGPTL4 (r_s_ = 0.514, p-adj = 5.30 × 10^− 4^), SAA with LBP (r_s_ = 0.600, p-adj = 2.48 × 10^− 5^) and ANGPTL4 (r_s_ = 0.539, p-adj = 2.68 × 10^− 4^). Significant positive correlations were observed also between SAA and CRP with CCL3, IL-15 and osteopontin. Positive correlations between acute phase proteins and measured analytes reflect the activation of the acute phase response and thus confirm the transcriptomic data. LBP correlated with neutrophil blood count (r_s_ = 0.327, *p* = 0.012), serum IgA concentration correlated with CXCL10 (r_s_ = 0.548, p-adj = 2.05 × 10^− 4^) and CCL19 (r_s_ = 0.549, p-adj = 2.04 × 10^− 4^) and serum albumin levels negatively correlated with MMP1 (r_s_ = -0.394, p-adj = 1.73 × 10^− 2^), osteopontin (rs = -0.474, p-adj = 2.10 × 10^− 3^), IL-15 (r_s_ = -0.482, p-adj = 1.57 × 10^− 3^) and ANGPTL4 (r_s_ = -0.482, p-adj = 1.57 × 10^− 3^). Significant positive correlations were observed between BMI and leptin (r_s_ = 0.620, p-ajd = 8.77 × 10^− 6^), FABP4 (r_s_ = 0.366, p-adj = 2.86 × 10^− 2^), IL-15 (r_s_ = 0.391, p-adj = 1.73 × 10^− 2^) and IL-18 (r_s_ = 0.370, *p* = 2.71 × 10^− 2^).

### Exploratory analysis of skin histopathological findings of IgAV patients and correlations with serum analytes

**Fig. 5:**
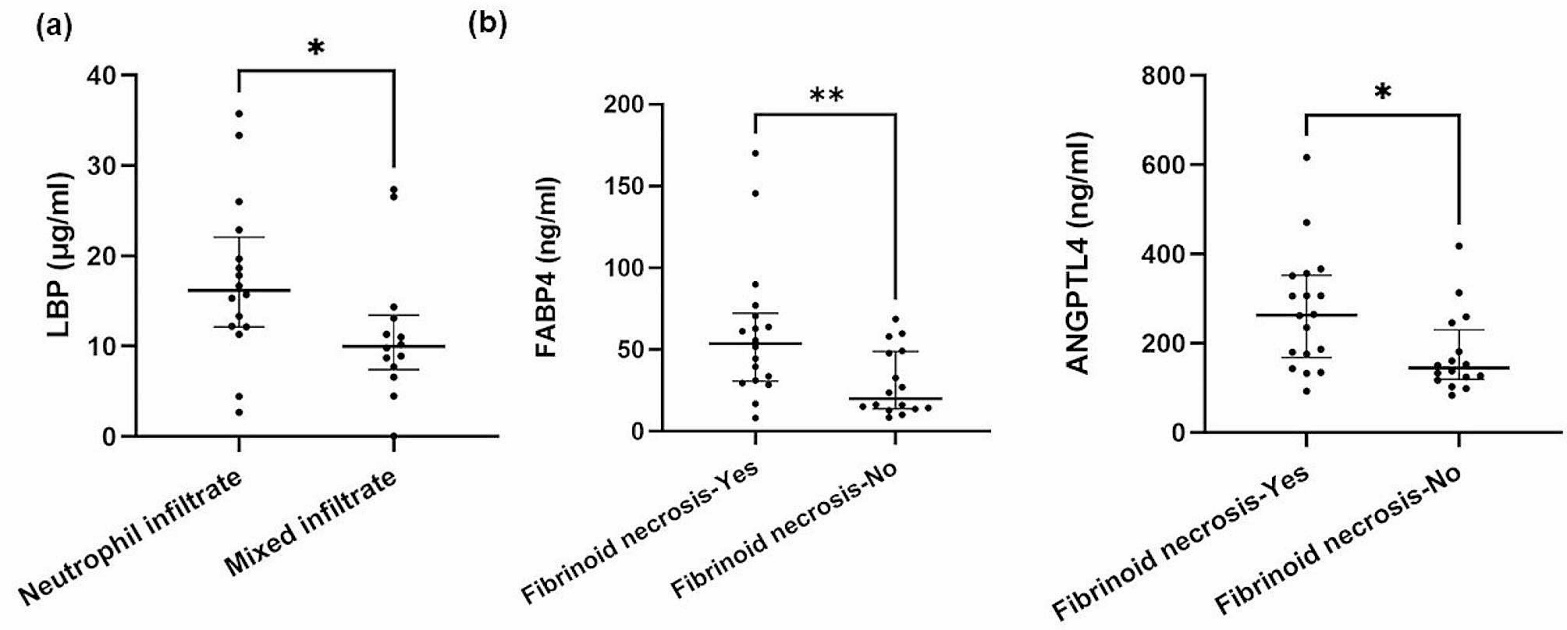
Associations of measured analytes with histopathological findings. **(a)** LBP serum level was significantly higher in patients with neutrophil infiltrates as compared to those with mixed infiltrates. **(b)** Patients with fibrinoid necrosis have increased serum FABP4 and ANGPTL4 levels as compared to those without. P-values were calculated using Mann Whitney U Test. Data are expressed as medians (Q25-Q75) of each group. *P ≤ 0.05, **P ≤ 0.01, ***P ≤ 0.001. IgAV, Immunoglobulin A vasculitis; CCL, Chemokine (C-C motif) ligand; ANGPTL4, angiopoietin-like 4; LBP, Lipopolysaccharide binding protein; FABP4, fatty acid-binding protein 4; ANGPTL4, angiopoietin-like 4

Skin histopathological findings from 34 patients were correlated with serum analytes. Inflammatory infiltrate, consisted of neutrophil granulocytes, mononuclear inflammatory cells or both (mixed infiltrate) were present in 14, 6 and 14 patients, respectively. Vessels with fibrinoid necrosis were found in 18 patients. Mild IgA deposits were present in 23 and abundant in 10 IgAV patients (Additional file 5: Table S4). IgA deposits were not determined in one patient. LBP serum level was higher (*p* = 0.035) in patients with neutrophil infiltrates as compared to those with mixed infiltrate (Fig. 5a). Patients with fibrinoid necrosis have increased serum FABP4 (*p* = 0.006) and ANGPTL4 (*p* = 0.014) levels as compared to those without (Fig. 5b). Patients with abundant IgA deposits had significantly elevated IL-15 (*p* = 2.96 × 10^− 2^) and CXCL10 (*p* = 3.82 × 10^− 2^) serum levels (Figure S7). After adjustment for multiple testing p-adj values did not reach statistical significance for reported exploratory analysis.

## Discussion

To our knowledge, this is the first study reporting an IgAV-associated skin mRNA signature in adults. Importantly, samples were collected from immunosuppressive treatment-naïve IgAV patients thus showing unmodulated pathological processes. PCA of skin transcriptome could distinguish between IgAV patients and HC. The IgAV patient closest to HC in the space spanned by the two most important PCs had a mild disease with nonnecrotic purpura and also low levels of inflammatory parameters (CRP, SAA and ESR), which might be the reason for the gene signature similar to controls. Differences observed in DEG in patients’ skin between sl-IgAV and IgAVN may indicate the activation of additional pathogenic processes in patients with systemic disease. The higher number of DEGs in IgAVN compared to sl-IgAV patients are not consequence of more severe skin involvement, as necrotic purpura was observed in only one patient from each group, while bullous purpura was also present in one sl-IgAV patient (Table S1). However, one of drawbacks of our study is lack of renal biopsies which were not clinically warranted. IgAVN diagnosis was set based on pathologic urinanalysis and improvement of renal parameters with immunomodulatory treatment.

Serum analytes measured in our study were selected in hypothesis free approach based on transcriptomic changes in the affected skin. Acute-phase response was one of the most highly enriched BP in IgAV skin, with the increased levels of acute-phase reactants, pro-inflammatory cytokines and chemokines. A recent study performing bidirectional Mendelian randomization analysis showed a causal effect of CRP, procalcitonin (PCT), and circulating inflammatory regulators on IgAV [19]. LBP was identified among the top five DEGs in IgAV skin and increased LBP was found also in patients’ sera. The levels of LBP correlated with SAA and CRP, however those two acute phase protein serum levels were not significantly changed in IgAVN as compared to sl-IgAV. Moreover machine learning approach identified LBP as the variable with the highest predictive value for IgAV. LBP was the only analyte in our study that was significantly increased in IgAVN compared to sl-IgAV patients, thus LBP could potentially be useful as a predictor/biomarker of renal involvement. Serum LBP level was also significantly increased in patients with IgA nephropathy, a separate disease that shares pathogenesis with IgAV [20]. During acute-phase response LBP binds to bacterial lipopolysaccharide (LPS) to elicit immune responses by presenting the LPS to cell surface pattern recognition receptors CD14 and Toll-like receptor (TLR) 4 [21]. Intriguingly, TLR4 expression was also increased in transcriptome of IgAVN patients. Blockade of the LPS-LBP interaction was a strategy to limit neutrophil infiltration in a mouse model of sepsis [22]. As positive correlation was observed between LBP and blood neutrophil count, and LBP was higher in patients with neutrophil infiltrates compared to those with mixed immune cells infiltrates, inhibition of LBP-TLR4 pathway might represent a potential therapeutic option also in IgAV [23].

Positive regulation of cytokine production and leukocyte mediated immunity are among enriched GO BP processes in IgAV skin. Due to their roles in leukocyte activation and migration, cytokines IL-15, IL-18, CCL3, CXCL5, CCL19 and CXCL10 were measured [24, 25]. IL-15, IL-18, CCL3 and CCL19 were increased in all IgAV patients compared to HC. Positive correlation was observed between serum IgA and IgA deposits in the skin with CCL19 and CXCL10. These two chemokines are type I Interferons (IFN)-regulated, associated with type I IFN signature in systemic lupus erythematosus (SLE) [26, 27]. CCL19 serum concentration was significantly increased in IgAV patients with necrotic compared to nonnecrotic purpura, suggesting that chemotactic activity might contribute to a more severe skin involvement.

Modifications in the skin’s ECM, identified through enriched KEGG pathways and GO CC, can be consequence of inflammation and tissue damage. Both measured ECM proteins MMP1 and osteopontin were elevated in IgAV sera compared to HC, with osteopontin among the analytes with the highest predictive value for IgAV.

Among the most highly enriched KEGG pathways identified in IgAV skin were Regulation of lipolysis in adipocytes and PPAR signalling pathway. Deregulations in lipid metabolism and adipokines are reported also in other skin and autoimmune diseases [28] as inflammatory response could lead to lipid and adipokine dysregulations [29]. Recent study reported alterations of serum lipids also in paediatric IgAV [30]. We found DEG of many adipokines, namely adiponectin, leptin, FABP4, SERPIN A12 and ANGPTL4 in IgAV skin and increased serum levels of leptin, FABP4 and ANGPTL4 in IgAV vs. HC. ANGPTL4 has shown a positive correlation with SAA and CRP and inverse correlation with serum albumin. In patients with more severe skin involvement, histologically presented as fibrinoid necrosis or with necrotic purpura we found higher ANGPTL4 levels than in nonnecrotic purpura. As ANGPTL4 is implicated in vascular inflammation and endothelial dysfunction further investigations of ANGPTL4 function in adult IgAV are warranted. Although serum adiponectin levels were not changed in all IgAV patients vs. HC, significantly lower levels were observed in IgAV_GI vs. sl-IgAV and IgAVN. The importance of adiponectin in predicting GI involvement was also confirmed with RF analysis in which adiponectin had a highest predictive value for GI involvement (Figure S4 and S5). As overweight and obesity are associated with decreased total adiponectin level [31], we performed linear regression analysis corrected for BMI, showing that difference still persists. Any imbalance of adiponectin from optimal serum levels might have detrimental effects, as indicated across various SARDs, including elevated levels in rheumatoid arthritis (RA) and SLE, and decreased levels in systemic sclerosis (SSc) [32].

Among the 15 measured analytes, levels of 11 analytes were significantly higher in treatment-naïve adult IgAV patients as compared to HC. As these differences could be attributed to age and sex as potential confounding factors, we performed linear regression analysis adjusted for age and sex still showing statistically significant differences between IgAV and HC. The performance of the RF and ST-EasyEnsemble algorithms in predicting IgAV using the measured analytes showed promising results, although it was difficult to predict visceral organ involvement. Nevertheless, we identified potential molecular markers of GI involvement, such as decreased adiponectin and CXCL10. Limitations of our study are the small number of patients included in the RNA sequencing and the potential influence of comorbidities on the measured analytes levels. In our study we compared IgAV skin transcriptomes with HC in order to identify deregulated processes, however comparison to other cutaneous vasculitis would be also informative for differential diagnostic purposes.

In conclusion, processes related to acute phase/inflammation, lipid metabolism and ECM are activated in IgAV adult skin lesions and are even more pronounced in patients with renal involvement. In our study, the most prominent biomarkers for predicting IgAV were LBP, ANGPT4 and osteopontin, involved in major deregulated processes, and associated with clinical characteristics. Furthermore, our study revealed new potential serum biomarkers of adult IgAV-related GI and renal involvement, namely adiponectin, CXCL10 and LBP.

## Conclusion

This is the first study to elucidate mRNA signatures of the affected skin from treatment-naïve adult IgAV patients. Transcriptomic analyses revealed perturbations in biological processes of acute phase response and lipid metabolism in patient’s skin and enabled IgAV patients’ subgroup differentiation. The genes implicated in deregulated biological processes were quantified in patient serum. Utilizing machine learning we were able to predict IgAV based on measured analytes, which could support clinicians in setting the diagnosis before histological results are available. Novel analytes were associated with clinical presentation, as LBP is increased in IgAV patients with renal involvement, while adiponectin and CXCL10 might be potential indicators of GI involvement in adult IgAV.

### Electronic supplementary material

Below is the link to the electronic supplementary material.

Supplementary Material 1

Supplementary Material 2

Supplementary Material 3

Supplementary Material 4

Supplementary Material 5

Supplementary Material 6

Supplementary Material 7

Supplementary Material 8

Supplementary Material 9

Supplementary Material 10

## Data Availability

All data has been uploaded to publicly available database (SRA#PRJNA1017657) and is also available by written request to the corresponding author.

## Abbreviations

ANGPTL4: angiopoietin-like 4
AUC: area under the ROC curve
BMI: body mass index
BP: biological processes
BVAS: Birmingham vasculitis activity score
CC: cellular components
CCL: chemokine (C-C motif) ligand
CI: confidence interval
CRP: C-reactive protein
CXCL: C-X-C motif chemokine ligand
DEG: differentially expressed gene
ECM: extracellular matrix
ESR: erythrocyte sedimentation rate
FABP4: fatty acid-binding protein 4
GI: gastrointestinal
GO: gene ontology
GSEA: gene set enrichment analysis
HC: healthy controls
IFN: Interferon
IgAV: immunoglobulin A vasculitis
IgAV-GI: IgAV-with gastrointestinal involvement
IgAVN: IgAV-with renal involvement
IgAVN + GI: IgAV-with renal and GI involvement
IL: interleukin
KEGG: Kyoto Encyclopedia of Genes and Genomes
LBP: lipopolysaccharide binding protein
LPS: lipopolysaccharide
MMP1: matrix metalloproteinase-1
NLR: neutrophil-to-lymphocyte ratio
ORA: over representation analysis
PC: principal component
PCA: principal component analysis
PCT: procalcitonin
PPAR: peroxisome proliferator-activated receptor
RA: rheumatoid arthritis
RF: random forest
ROC: receiver operating characteristic
SAA: serum amyloid A
SARD: systemic autoimmune rheumatic disease
SLE: systemic lupus erythematosus
sl-IgAV: skin-limited IgAV
SSc: systemic sclerosis
TLR: toll-like receptor
TNF: tumor necrosis factor

## References

[1] Jennette, Falk, Bacon, Basu N, Cid, Ferrario (2013). 2012 revised international chapel Hill Consensus Conference nomenclature of Vasculitides. Arthritis Rheum.

[2] Hetland LE, Susrud KS, Lindahl KH, Bygum A (2017). Henoch-Schönlein Purpura: A literature review. Acta Derm Venereol.

[3] Carlson JA (2010). The histological assessment of cutaneous vasculitis. Histopathology.

[4] Hočevar A, Tomšič M, Pižem J, Bolha L, Sodin-Šemrl S, Glavač D (2019). MicroRNA expression in the affected skin of adult patients with IgA vasculitis. Clin Rheumatol.

[5] Jurčić VBL, Matjašič A, Sedej I, Dolinar A, Grubelnik G, Hauptman N, Pižem J, Jevšinek-Skok D, Hočevar A, Ravnik-Glavač M, Glavač D (2019). Association between histopathological changes and expression of selected microRNAs in skin of adult patients with IgA vasculitis. Histopathology.

[6] Piram M, Mahr A (2013). Epidemiology of immunoglobulin A vasculitis (Henoch-Schonlein): current state of knowledge. Curr Opin Rheumatol.

[7] Audemard-Verger APE, Baldolli A, Gouellec NL, Augusto JF, Jourde-Chiche N, Raffray L, Thervet E, Deroux A, Goutte J, Hummel A, Lioger B, Sanges S, Cacoub P, Amoura Z, Moulis G, Maurier F, Lavigne C, Urbanski G, Chanal J, Faguer S, Deriaz S, Feirreira-Maldent N, Diot E, Maillot F, Guillevin L, Terrier B (2021). Impact of aging on phenotype and prognosis in IgA vasculitis. Rheumatology (Oxford).

[8] Hocevar A, Tomsic M, Jurcic V, Perdan Pirkmajer K, Rotar Z (2019). Predicting gastrointestinal and renal involvement in adult IgA vasculitis. Arthritis Res Therapy.

[9] Kuret T, Lakota K, Zigon P, Ogric M, Sodin-Semrl S, Cucnik S (2019). Insight into inflammatory cell and cytokine profiles in adult IgA vasculitis. Clin Rheumatol.

[10] Kawakami T, Watabe H, Mizoguchi M, Soma Y (2006). Elevated serum IgA anticardiolipin antibody levels in adult Henoch-Schönlein purpura. Br J Dermatol.

[11] Delapierre A, Terrier B, Pillebout E, Baudart P, Jourde-Chiche N, Lioger B (2022). Clinical phenotype and cytokine profile of adult IgA vasculitis with joint involvement. Clin Rheumatol.

[12] Audemard-Verger A, Pillebout E, Jamin A, Berthelot L, Aufray C, Martin B (2019). Recruitment of CXCR3(+) T cells into injured tissues in adult IgA vasculitis patients correlates with disease activity. J Autoimmun.

[13] Hočevar A, Rotar Ž, Jurčić V, Čučnik S, Tomšič M (2015). Patient age, gender and extent of purpura may suggest short-term outcomes in adults with IgA vasculitis. Rheumatology (Oxford).

[14] Ozen S, Pistorio A, Iusan SM, Bakkaloglu A, Herlin T, Brik R (2010). EULAR/PRINTO/PRES criteria for Henoch-Schonlein purpura, childhood polyarteritis nodosa, childhood Wegener granulomatosis and childhood Takayasu arteritis: Ankara 2008. Part II: final classification criteria. Ann Rheum Dis.

[15] Zhu A, Ibrahim JG, Love MI (2018). Heavy-tailed prior distributions for sequence count data: removing the noise and preserving large differences. Bioinformatics.

[16] Blagus R, Lusa L (2017). Gradient boosting for high-dimensional prediction of rare events. Comput Stat Data Anal.

[17] Breiman L (2001). Random forests. Mach Learn.

[18] Team RC. R: A language and environment for statistical computing. Vienna, Austria: R Foundation for Statistical Computing 2021 [ https://www.R-project.org/.

[19] Qin J, Zhang L, Ke B, Liu T, Kong C, Jin C. Causal relationships between circulating inflammatory factors and IgA vasculitis: a bidirectional mendelian randomization study. Front Immunol. 2023;14. 10.3389/fimmu.2023.1248325 PMC10518517 37753071

[20] Zhong Z, Tan J, Tan L, Tang Y, Qiu Z, Pei G (2020). Modifications of gut microbiota are associated with the severity of IgA nephropathy in the Chinese population. Int Immunopharmacol.

[21] Ding PH, Jin LJ (2014). The role of lipopolysaccharide-binding protein in innate immunity: a revisit and its relevance to oral/periodontal health. J Periodontal Res.

[22] Fang H, Hua C, Weiss S, Liu A, Cheng W, Claus R (2018). Modulation of Innate immunity by G-CSF and inflammatory response by LBPK95A improves the outcome of Sepsis in a rat model. J Immunol Res.

[23] Chen X-Q, Tu L, Tang Q, Huang L, Qin Y-H. An emerging role for Neutrophil Extracellular traps in IgA Vasculitis: a Mini-review. Front Immunol. 2022;13. 10.3389/fimmu.2022.912929 PMC9253285 35799774

[24] Patidar M, Yadav N, Dalai SK (2016). Interleukin 15: a key cytokine for immunotherapy. Cytokine Growth Factor Rev.

[25] Yang YL, Li XF, Song B, Wu S, Wu YY, Huang C (2023). The role of CCL3 in the pathogenesis of rheumatoid arthritis. Rheumatol Ther.

[26] Enocsson H, Wetterö J, Eloranta ML, Gullstrand B, Svanberg C, Larsson M (2021). Comparison of surrogate markers of the type I Interferon Response and their ability to Mirror Disease activity in systemic Lupus Erythematosus. Front Immunol.

[27] Bauer JW, Petri M, Batliwalla FM, Koeuth T, Wilson J, Slattery C (2009). Interferon-regulated chemokines as biomarkers of systemic lupus erythematosus disease activity: a validation study. Arthritis Rheum.

[28] Kovács D, Fazekas F, Oláh A, Törőcsik D. Adipokines in the skin and in Dermatological diseases. Int J Mol Sci. 2020;21(23). 10.3390/ijms21239048 PMC7730960 33260746

[29] Gibson MS, Domingues N, Vieira OV (2018). Lipid and non-lipid factors affecting macrophage dysfunction and inflammation in atherosclerosis. Front Physiol.

[30] Liu Y, Wen M, He Q, Dang X, Feng S, Liu T (2022). Lipid metabolism contribute to the pathogenesis of IgA Vasculitis. Diagn Pathol.

[31] Gariballa S, Alkaabi J, Yasin J, Al Essa A (2019). Total adiponectin in overweight and obese subjects and its response to visceral fat loss. BMC Endocr Disorders.

[32] Brezovec N, Perdan-Pirkmajer K, Čučnik S, Sodin-Šemrl S, Varga J, Lakota K. Adiponectin Deregulation in systemic Autoimmune Rheumatic diseases. Int J Mol Sci. 2021;22(8). 10.3390/ijms22084095 PMC8071452 33920997

