# Supplementary material for: Deregulation in adult IgA vasculitis skin as the basis for the discovery of novel serum biomarkers
Source: Arthritis Res Ther. 2024 Apr 12;26:85. doi: 10.1186/s13075-024-03317-6 (PMC11010360; doi:10.1186/s13075-024-03317-6)
Supplement: Supplementary file 1 — Supplementary Material 1 [file 13075_2024_3317_MOESM1_ESM.docx]

**Table S3.** Number of DEGs (|log2(FC)|≥1 and p-adjusted value (p-adj) ≤ 0.05) in IgAV patients vs healthy controls (HC), IgAVN vs HC, sl-IgAV vs HC and IgAVN vs sl-IgAV

| **Comparison (A vs B)** | Number DEGs | Up-regulated in A | Down-regulated in A |
| --- | --- | --- | --- |
| IgAV vs HC | 49 | 47 | 2 |
| IgAVN vs HC | 507 | 325 | 182 |
| sl-IgAV vs HC | 46 | 30 | 16 |
| IgAVN vs sl-IgAV | 136 | 99 | 37 |

IgAV, immunoglobulin A vasculitis; IgAVN, IgAV-renal involvement, IgAV_S, IgAV-skin-limited disease; HC, healthy controls; DEGs, differentially expressed genes.
