# Supplementary material for: Deregulation in adult IgA vasculitis skin as the basis for the discovery of novel serum biomarkers
Source: Arthritis Res Ther. 2024 Apr 12;26:85. doi: 10.1186/s13075-024-03317-6 (PMC11010360; doi:10.1186/s13075-024-03317-6)
Supplement: Supplementary file 2 — Supplementary Material 2 [file 13075_2024_3317_MOESM2_ESM.docx]

**Table S3.** Skin histopathological changes in 34 IgAV patients

| Histopathological change | Evaluation | Number of patients |
| --- | --- | --- |
| Composition of immune cell infiltrate |  |  |
|  | Neutrophils | 14 |
|  | Mononuclear inflammatory cells | 6 |
|  | Mixed infiltrate | 14 |
| Presence of fibrioid necrosis |  | 18 |
| Immunoglobulin A |  |  |
|  | Mild | 23 |
|  | Abundant | 10 |
