# Supplementary material for: Deregulation in adult IgA vasculitis skin as the basis for the discovery of novel serum biomarkers
Source: Arthritis Res Ther. 2024 Apr 12;26:85. doi: 10.1186/s13075-024-03317-6 (PMC11010360; doi:10.1186/s13075-024-03317-6)
Supplement: Supplementary file 8 — Supplementary Material 8 [file 13075_2024_3317_MOESM8_ESM.docx]

**Table S1** Demographic and clinical characteristics of IgAV patients, IgAVN, sl-IgAV and HC included in RNA sequencing

| **Characteristics** | **IgAV (N=6)** | **IgAVN (N=3)** | **sl-IgAV (N=3)** | **HC (N=3)** |
| --- | --- | --- | --- | --- |
| Age* | 66.5 (56.6-76.7) | 66.6 (66.3-74.2) | 64 (34.2-84.2) | 53 (48-57.5) |
| Sex | 1M, 5F | 3F | 1M, 2F | 3F |
| BMI* | 30.4 (24.6-35.9) | 31.8 (26-40) | 29.1 (20.4-34.5) | / |
| Symptom duration (day)* | 5.5 (3.5-38) | 5 (2-14) | 6 (4-110) | / |
| Skin purpura | 6 | 3 | 3 | / |
| Purpura above waistline | 4 | 2 | 2 | / |
| CRP (g/l) | 51.5 (27.8-135.8) | 58 (32-198) | 45 (15-115) | / |
| SAA (μg/ml) | 38.4 (0-175) | 302 (92.5-933) | 38.4 (0.00-598.0) | / |
| Number of lymphocytes (10^9^/l) | 1.83 (1.08-2.62) | 1.58 (0.75-2.57) | 2.08 (1.19-2.76) | / |
| Number of neutrophils (10^9^/l) | 5.12 (4.12-6.19) | 4.5 (4.47-6.94) | 7.46 (4.31-10.44) | / |
| Skin necroses | 2 | 1 | 1 | / |
| Bullous skin lesions | 1 | 0 | 1 | / |
| BVAS* | 7 (2-11) | 8 (8-20) | 2 (2-6) | / |
| Concurrent infection | 1 | 0 | 1 | / |
| Prior infection | 2 | 1 | 1 | / |

IgAV, Immunoglobulin A vasculitis; IgAVN, IgAV with renal involvement; sl-IgAV, IgAV-skin-limited disease; HC, healthy controls; M, male; F, female; BVAS, Birmingham vasculitis activity score; * median (IQR); BMI body mass index
