# Supplementary material for: Deregulation in adult IgA vasculitis skin as the basis for the discovery of novel serum biomarkers
Source: Arthritis Res Ther. 2024 Apr 12;26:85. doi: 10.1186/s13075-024-03317-6 (PMC11010360; doi:10.1186/s13075-024-03317-6)

**Figure S1** Enriched KEGG pathways, GO BP and CC in IgAV patients vs HC, IgAVN vs HC, sl-IgAV vs HC and IgAVN vs sl-IgAV as identified with gene set enrichement analysis (GSEA). KEGG, Kyoto Encyclopedia of Genes and Genomes; GO, Gene Ontology; BP, Biological Processes; CC, Cellular Components; IgAV, immunoglobulin A vasculitis; HC, healthy controls; IgAVN, IgAV with renal involvement; sl-IgAV, skin-limited IgAV.


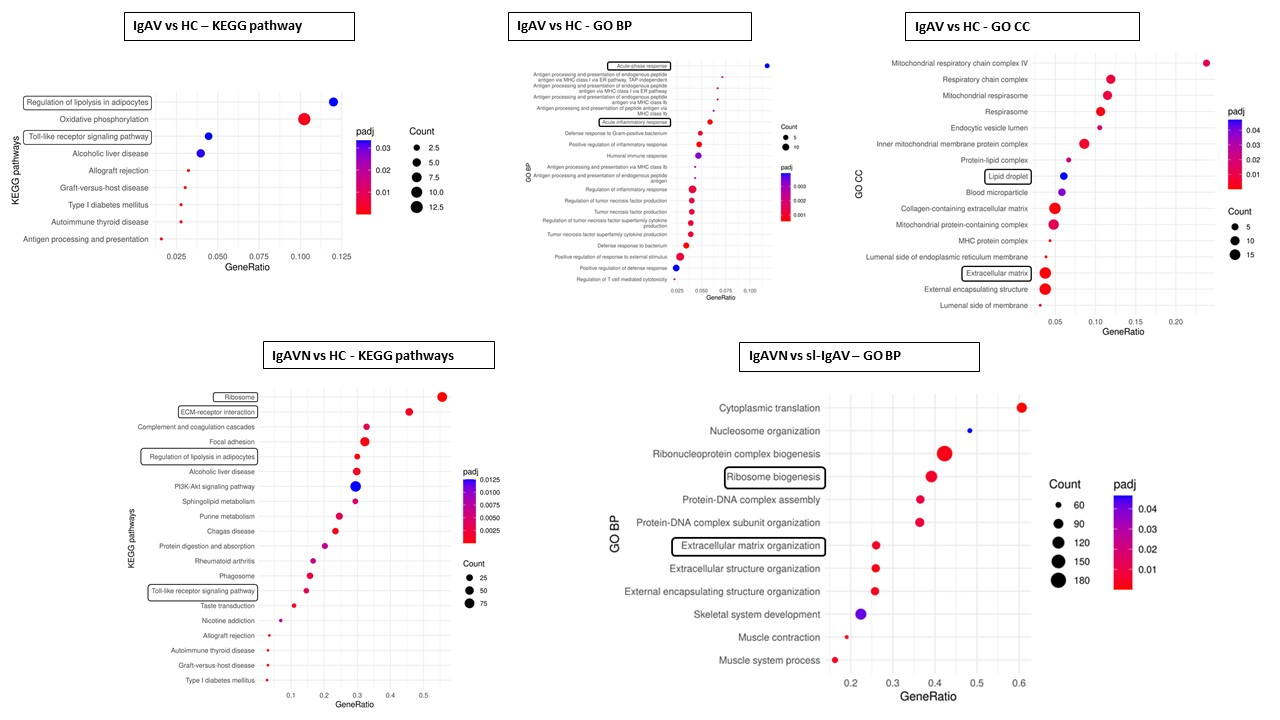


**Figure S2** Heat map representing gene expression patterns in IgAVN patients and HC, and the corresponding proteins were measured in patients' sera to study their implications in dysregulated biological processes observed in the patients' skin. Each row corresponds to a specific gene, and each column represents an individual sample. The colours in the heatmap reflect the levels of gene expression, with darker colours indicating higher expression and brighter colours indicating lower expression. Z-score represents the standard deviation by which gene expression values deviate from the mean value. IgAV, Immunoglobulin A vasculitis; IgAVN, IgAV with renal involvement; HC, healthy controls; LBP, Lipopolysaccharide binding protein; AdipoQ, Adiponectin; SPP1, osteopontin; FABP4, fatty acid-binding protein 4; ANGPTL4, angiopoietin-like 4; LEP, leptin; KLK5, kallikrein 5.


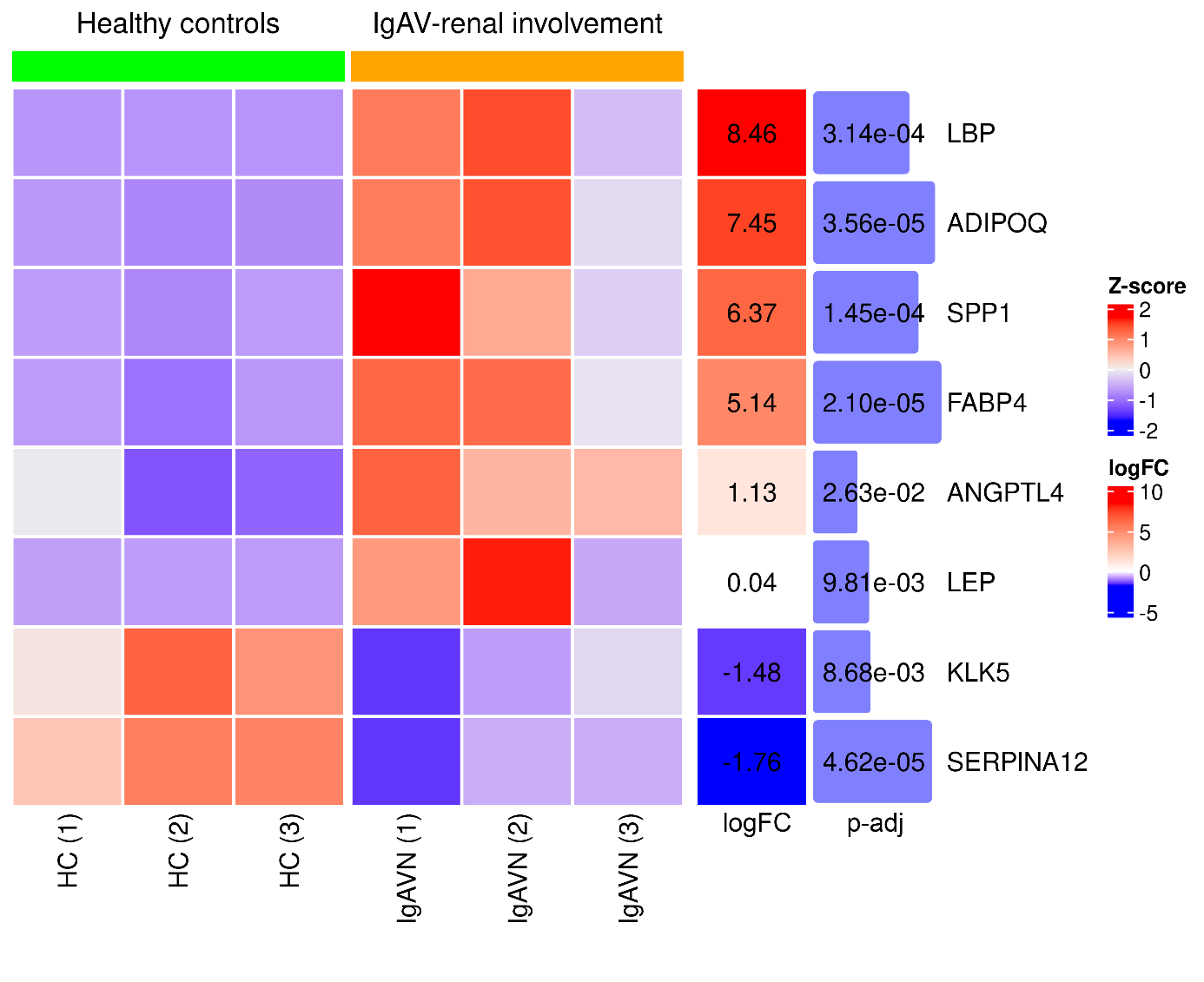


**Figure S3** Classification of adult IgAV using St_EE model **(a)** ROC curve of St_EE with corresponding confidence interval. Red line presents ROC curve of random classificator (AUC = 0.5). **(b)** First ten variables according to their relative importance for prediction of adult IgAV. IgAV, Immunoglobulin A vasculitis; ROC, Receiver operating characteristic; AUC, area under the curve; St_EE, ST-EasyEnsemble; LBP, Lipopolysaccharide binding protein; CXCL, C-X-C motif chemokine ligand; ANGPTL4, angiopoietin-like 4; FABP4, fatty acid-binding protein 4; IL, interleukin.


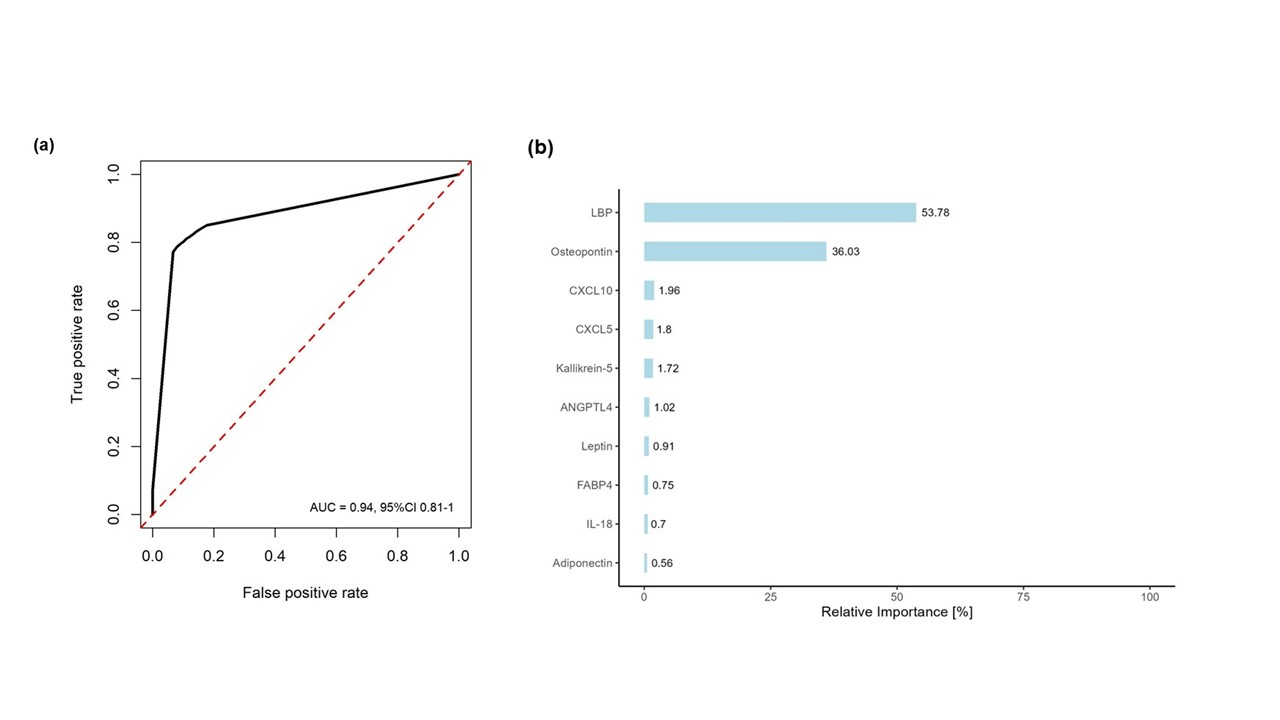


**Figure S4** The potential of measured analytes for predicting GI involvement **(a)** PCA of log2 transformed serum analyte concentrations distinguished IgAV_GI&IgAVN+GI from IgAV_NO_GI. **(b)** ROC curve of Random forest algorithm with corresponding confidence interval for predicting GI involvement. Red line presents ROC curve of random classificator (AUC = 0.5). **(c)** First ten variables according to their relative importance for prediction of GI involvement. IgAV, Immunoglobulin A vasculitis; PCA, principal component analysis; IgAVN, IgAV with renal involvement; IgAV_GI, IgAV with gastrointestinal involvement (GI); IgAVN+GI, IgAV with GI and renal involvement; ROC, Receiver operating characteristic; AUC, area under the curve; CXCL, C-X-C motif chemokine ligand; CCL, Chemokine (C-C motif) ligand; LBP, lipopolysaccharide binding protein; IL, interleukin; MMP, matrix metalloproteinase; FABP4, fatty acid-binding protein 4.


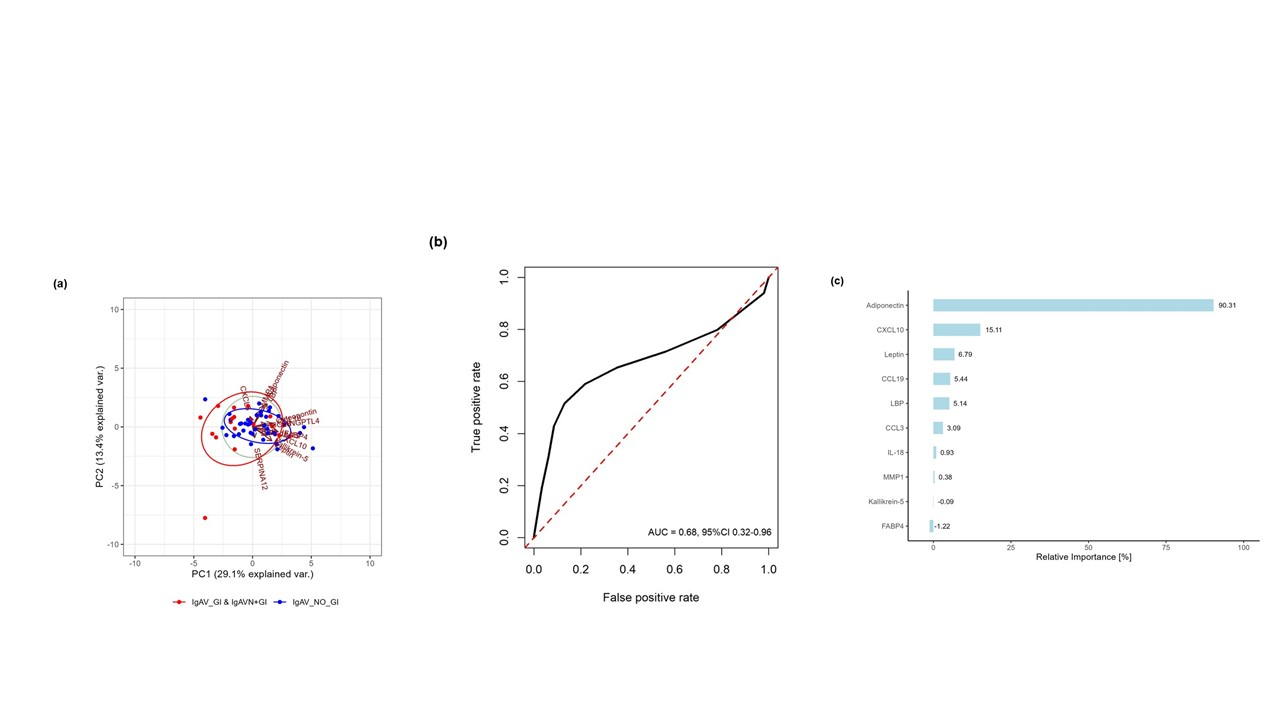


**Figure S5** The potential of measured analytes for predicting GI involvement assessed by St_EE model **(a)** ROC curve of St_EE model with corresponding confidence interval for predicting GI involvement. Red line presents ROC curve of random classificator (AUC = 0.5). **(b)** First ten variables according to their relative importance for prediction of GI involvement. IgAV, Immunoglobulin A vasculitis; GI, gastrointestinal; ROC, Receiver operating characteristic; AUC, area under the curve; St_EE, ST-EasyEnsemble; MMP, matrix metalloproteinase; ANGPTL4, angiopoietin-like 4; CCL, Chemokine (C-C motif) ligand; CXCL, C-X-C motif chemokine ligand; LBP, lipopolysaccharide binding protein; IL, interleukin.


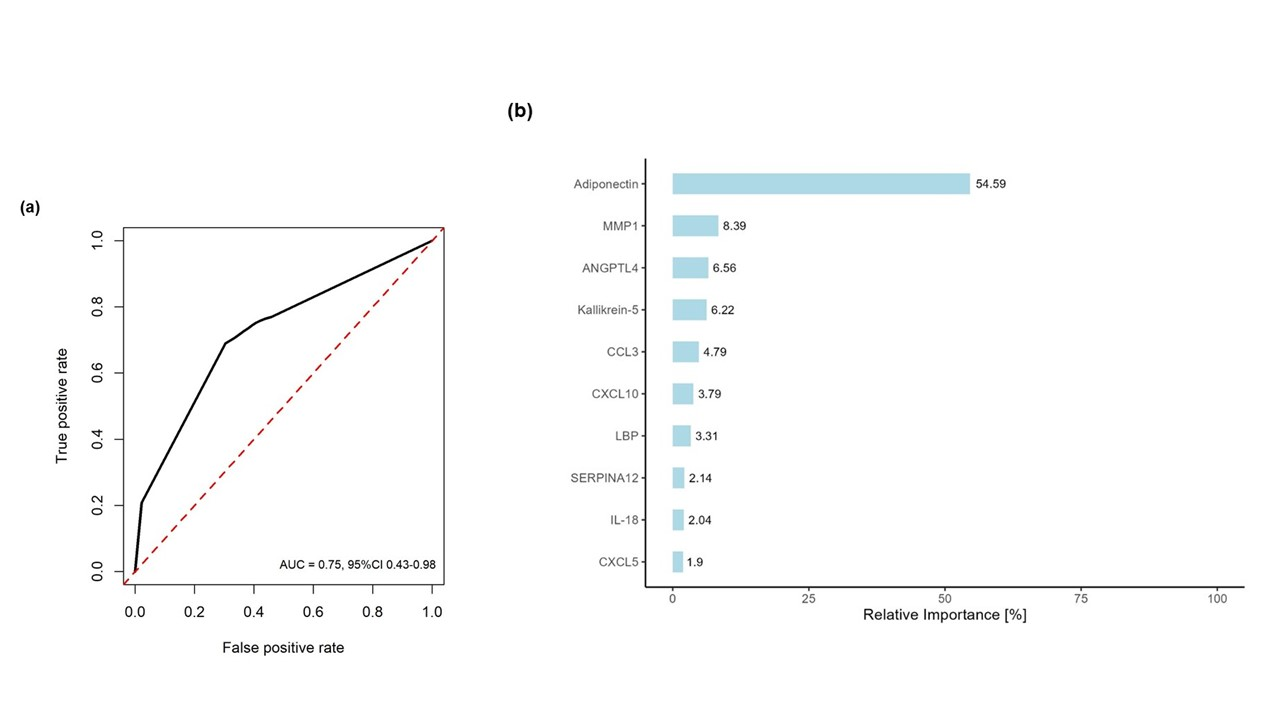


**Figure S6** Correlation matrix heatmap including 15 selected analytes, routinely measured immunological parameters (antibody levels, white blood cell count, inflammatory markers), age, BMI and BVAS in 59 IgAV patients. Only correlation coefficients with P-values < 0.05 are labelled. FABP4, fatty acid-binding protein 4; IL, interleukin; ANGPTL4, angiopoietin-like 4; CXCL, C-X-C motif chemokine ligand; CCL, Chemokine (C-C motif) ligand; MMP, matrix metalloproteinase; LBP, lipopolysaccharide binding protein; SAA, serum amyloid A; Ig, immunoglobulin; NLR, neutrophil-to-lymphocyte ratio; LYM, lymphocytes; NEUT, neutrophils; WBCs, White blood cells; CRP, C-reactive protein; ESR, Erythrocyte sedimentation rate; BVAS, Birmingham vasculitis activity score; BMI, body mass index.


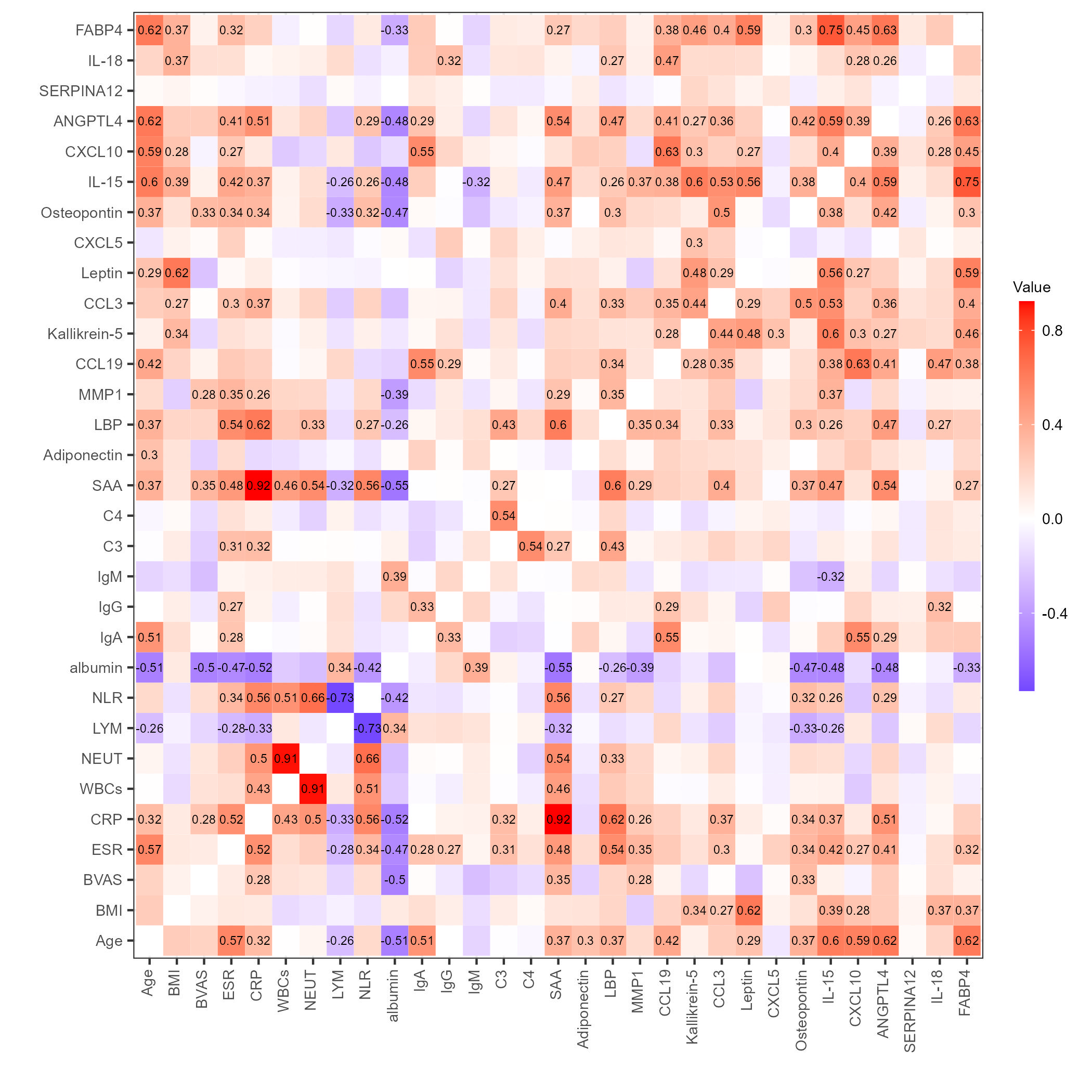


**Figure S7** Point-biserial correlations between skin histopathological changes and 15 selected analytes and BVAS disease activity score in 34 IgAV patients. The brackets denote the compared variables, while the square brackets denote reference variable. FABP4, fatty acid-binding protein 4; IL, interleukin; ANGPTL4, angiopoietin-like 4; CXCL, C-X-C motif chemokine ligand; CCL, Chemokine (C-C motif) ligand; MMP, matrix metalloproteinase; LBP, lipopolysaccharide binding protein; Ig, immunoglobulin; NEUT, neutrophils; BVAS, Birmingham vasculitis activity score; MONO, Mononuclear inflammatory cells.


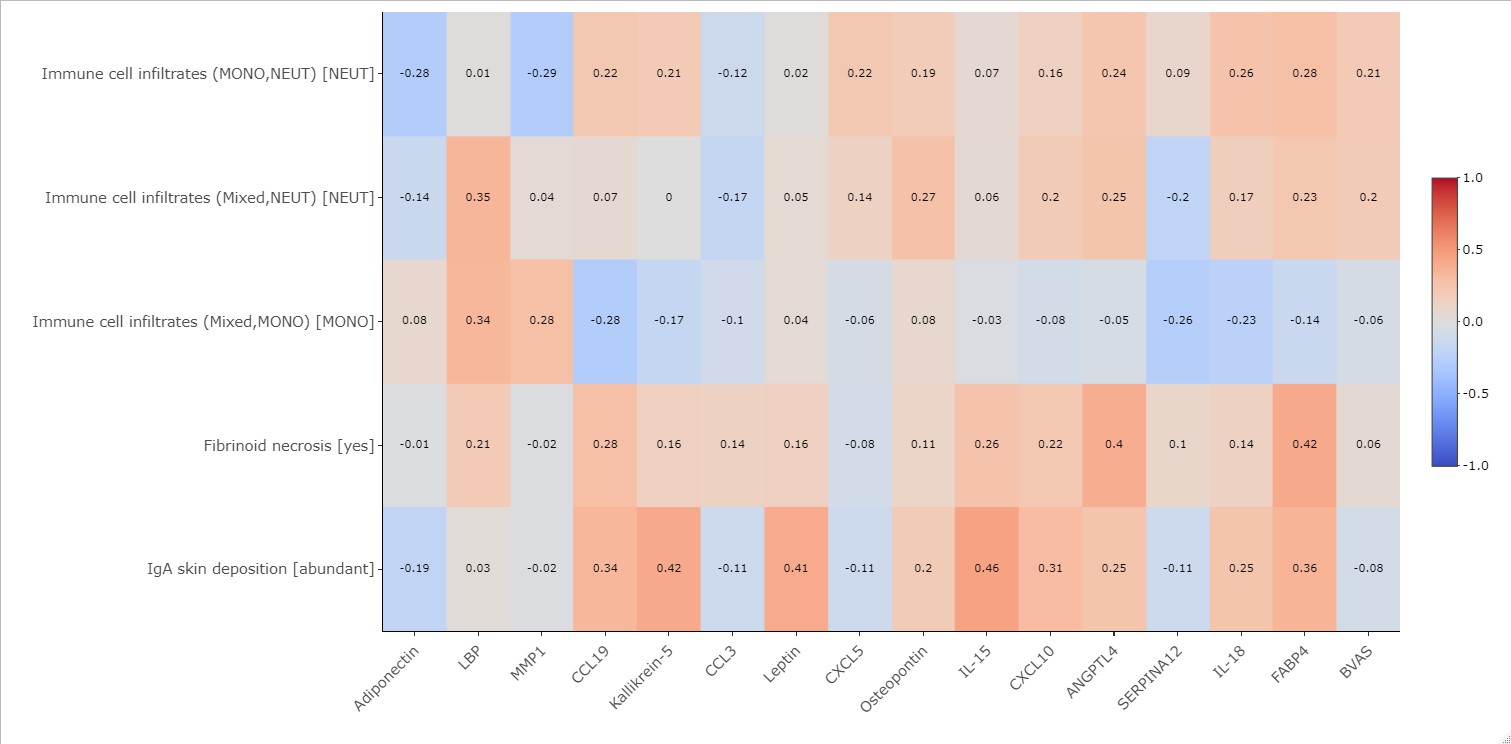

Supplement: Supplementary file 9 — Supplementary Material 9 [file 13075_2024_3317_MOESM9_ESM.docx]
