# Supplementary material for: Deregulation in adult IgA vasculitis skin as the basis for the discovery of novel serum biomarkers
Source: Arthritis Res Ther. 2024 Apr 12;26:85. doi: 10.1186/s13075-024-03317-6 (PMC11010360; doi:10.1186/s13075-024-03317-6)
Supplement: Supplementary file 10 — Supplementary Material 10 [file 13075_2024_3317_MOESM10_ESM.docx]

**Table S2** Demographic, laboratory and clinical characteristics of IgAV patients and healthy controls HC included in serum analyte measurement

| **Characteristics** | **IgAV (N=59)** | **HC (N=22)** |
| --- | --- | --- |
| Age | 64.8 (49.4-70.3) | 58.7 (56.4-62.0) |
| Sex | 41 M, 18 F | 17 M, 5 F |
| BMI* | 29.2 (24.9-32.6) | / |
| Symptom duration (day)* | 7 (5-14) | / |
| Symptoms and signs N (%) | | |
| General symptoms | 11 (18.3) | / |
| Fever | 4 (6.8) | / |
| Weight loss | 8 (13.6) | / |
| Skin purpura | 59 (100) | / |
| Purpura above waistline | 38 (66.1) | / |
| Skin necroses | 30 (50.8) | / |
| Joint involvement | 8 (13.6) |  |
| GI involvement | 19 (32.2) | / |
| Renal involvement | 31 (52.5) | / |
| Concurrent infection | 11 (18.6) | / |
| Prior infection | 19 (32.2) | / |
| BVAS* (Q25–Q75) | 9 (2-14) | / |
|  | | |
| ESR* (mm/h) | 34 (14-53) | / |
| CRP* (g/l) | 21 (7-58) | / |
| SAA* (µg/ml) | 39 (4.8-175.3) | / |
| Serum albumin (g/l) | 36 (31-41) |  |
| White cells* (10^9^/l) | 8.9 (7.4-10.3) | / |
| Number of lymphocytes (10^9^/l) | 1.58 (1.1-1.99) | / |
| Number of neutrophils (10^9^/l) | 6.1 (4.9-7.9) | / |
| IgA* (g/l) | 3.98 (2.63-5.32) | / |
| IgG* (g/l) | 12.3 (9.87-14.89 | / |
| IgM* (g/l) | 0.75 (0.4-1.07) | / |
| C3* (g/l) | 1.35 (1.19-1.5) | / |
| C4* (g/l) | 0.28 (0.23-0.33) | / |

IgAV, Immunoglobulin A vasculitis; HC, healthy controls; M, male; F, female; BVAS, Birmingham vasculitis activity score; ESR, Erythrocyte sedimentation rate; CRP, C-reactive protein (CRP); SAA, Serum Amyloid A; Ig, immunoglobulin; * median (IQR); BMI body mass index
